# Atomistic Modeling of Cross-Linking in Epoxy-Amine Resins: An Open-Source Protocol

**DOI:** 10.1021/acsapm.4c04208

**Published:** 2025-04-03

**Authors:** Marina Provenzano, Francesco Maria Bellussi, Matteo Fasano, Hernán Chávez Thielemann

**Affiliations:** † Department of Energy, Politecnico di Torino, Corso Duca degli Abruzzi 24, 10129 Torino, Italy; ‡ Department of Mechanical Engineering, Eindhoven University of Technology, 5612 AZ Eindhoven, The Netherlands

**Keywords:** atomistic modeling, epoxy resins, polymeric materials, molecular dynamics simulations, thermo-mechanical properties, cross-linking process

## Abstract

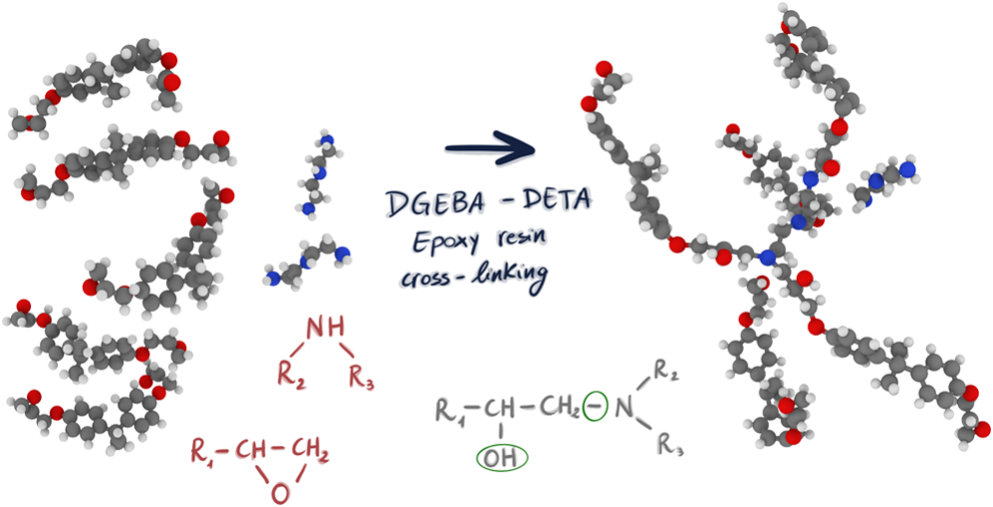

Atomistic modeling has become an extensively used method for studying thermosetting polymers, particularly in the analysis and development of high-performance composite materials. Despite extensive research on the topic, a widely accepted, standardized, flexible, and open-source approach for simulating the cross-linking process from precursor molecules has yet to be established. This study proposes, tests, and validates a Molecular Dynamics (MD) protocol to simulate the cross-linking process of epoxy resins. We developed an in-house code based on Python and LAMMPS, enabling the generation of epoxy resin structures with high degrees of cross-linking. In our work, the epoxy network is dynamically formed within the MD simulations, modeling the chemical bonding process with constraints based on the distance between the reactive sites. To validate our model against experimental data from the literature, we then computed the density, thermal conductivity, and elastic response. The results show that the produced structures align well with experimental evidence, validating our method and confirming its feasibility for further analyses and in silico experiments. Beyond the case study presented in this work, focusing on bisphenol A diglycidyl ether (DGEBA) epoxy resin and diethylenetriamine (DETA) as curing agents in a 5:2 ratio, our approach can be easily adapted to investigate different epoxy resins.

## 1 Introduction

The study and characterization of thermosetting polymers currently play a significant role in both academia and industry due to their importance in the development of advanced composite materials, as well as the relevance of their disposal in the context of creating a circular and sustainable economy.^1^ Thermosets are obtained by an irreversible reaction that occurs between a prepolymer (resin) and a curing agent (hardener). The curing reaction results in the creation of a network of molecules linked by chemical bonds. As a result, these polymers exhibit superior properties compared to most thermoplastic polymers, including enhanced creep resistance, hardness, thermo-dimensional stability, and increased compressive strength.^2,3^ Epoxy resins, in particular, are versatile and high-performance thermosetting polymers that are extensively utilized in coatings, structural adhesives, and composite matrices. Their importance is particularly notable in fiber-reinforced composites in the aerospace and defense sectors due to their stable mechanical properties and excellent chemical compatibility with a wide range of fillers.^4,5^

Over the past two decades, molecular dynamics (MD) simulations have played a pivotal role in determining the properties of various systems.^6−11^ This approach offers a cost-effective way to evaluate the performance and behavior of materials, significantly reducing the effort and time associated with traditional experimental error and testing methods. MD simulations are valued for their reasonable accuracy, repeatability, and flexibility in adapting to different analyses, and in the case of epoxy resin, they allow for a thorough understanding of the network structure and its impact on thermo-mechanical properties.^12^ Accurately developing the cross-linked structure of thermoset materials, however, is a challenging task as it requires simulation of the curing process and ongoing updating of the molecular topology.

Numerous researchers have employed commercial software to generate cross-linked resin samples before conducting simulations of physical observables.^13,14^ More recently, the introduction of reactive force fields (Reax-FF^15^) has enabled the direct study of the cross-linking process,^16^ but these methodologies often require time-intensive iterations to optimize the force field for specific molecular systems. Moreover, simulations using reactive force fields are computationally very expensive compared with the use of classical force fields. Vashisth et al. and Radue et al. proposed an accelerated reactive force field capable of constructing an epoxy network with high cross-link conversion,^17,18^ Nishino et al. created different models of bulk epoxy resin, trying both DREIDING^19^ force field and Reax-FF,^15,20^ while Karuth et al. applied the Reax-FF potential to obtain an epoxy-amine network.^21^ On the other hand, Odegard et al. have recently modeled epoxy resin using the reactive interface force field,^22,23^ while Oya et al. used quantum chemical calculations coupled with MD simulations.^24^

Other authors have developed alternative methods based on several classical and well-established force fields, such as DREIDING,^19,25^ CHARMM,^26,27^ COMPASS,^28,29^ and AMBER,^30,31^ to simulate thermosetting polymer systems and successfully reproducing their structures and physical properties without requiring the use of the computationally demanding reactive force fields.^32−34^ Orselly et al., for example, implemented a multistep cross-linking algorithm and performed all-atom molecular dynamics simulations with the CHARMM force field to characterize various epoxy resins.^27^ In classical MD methods, a stopping criterion is generally introduced for the cross-linking process based on a maximum distance between potentially reactive sites or a target number of created bonds (thus a certain cross-linking degree is achieved). Moreover, these pseudoreactive methodologies allow for a dynamic simulation of the reaction, which offers the advantage of taking into account the presence of any molecules other than resin precursors, such as nanofillers, and thus allowing the simulation of the properties of composite materials.^35−38^

However, a widely accepted, standardized, flexible, and open-source method for simulating the cross-linking process with MD simulations has yet to be established. This work introduces a protocol for modeling the highly cross-linked epoxy matrix and validates its efficacy through a test scenario involving an epoxy-amine mixture with material properties compared with experimental data available in the literature. In detail, the goal of this work is to create a flexible protocol that can be directly acted upon as needed, with the ultimate prospect of simulating not only the creation of the epoxy resin network but also its decomposition in recycling processes such as solvolysis.^39−42^

## 2 Methods

The methodology adopted in this work can be divided into three main parts to ensure a thorough understanding, efficient handling, and automation of each step. The first step involves studying the molecular system of interest, including data on precursors and other molecules, selecting a force field capable of representing all interactions, and building a DGEBA-DETA mixture, i.e., a simulation box with a specific precursor ratio for the reaction. The second part consists of implementing the curing reaction, in which the reactive sites of the molecules form chemical bonds, resulting in a polymer network. The system obtained can then be relaxed to ambient conditions. Finally, the cured polymer system is characterized by evaluating some thermo-mechanical properties, and numerical results are compared with experimental data available in the literature.

The structures are initially built with Packmol^43^ and visualized in OVITO,^44^ to verify the topology at various stages of the process. The assignment and management of force field parameters is accomplished by VMD^45^ and self-written codes in Python.^46^ All MD simulations are performed with LAMMPS,^47−50^ with all codes available open source in a Zenodo archive.^51^ The following subsections are intended to explain the protocol used, with respect to the assumptions made and the details of the simulations.

### 2.1 Studied Thermosetting Resin

**Figure 1:**
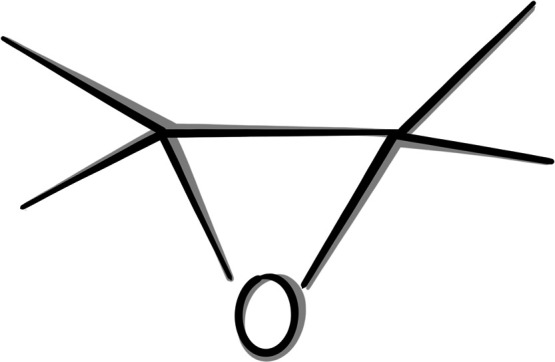
1,2-Epoxide group.

Epoxy resins are characterized by a remarkably high number of epoxy groups (Figure 1), which are structures consisting of a ring made up of an oxygen atom and two carbon atoms.^52^ Market-relevant epoxy resins contain two or more epoxide groups that can easily react with some other compounds called curing agents, which have multiple reactive sites, such as those found in amines. The widely used name for this polymerization process is cross-linking, which involves the creation of a network of resin and curing agent molecules bound to each other in a solid structure. In general, curing agents with a functionality greater than 2 are used to guarantee the formation of a polymer network. The high stability and partial tunability have made epoxy resin materials suitable for a wide range of applications.^53^ In this work, we focus on the industrially relevant case study of bisphenol A diglycidyl ether epoxy resin (DGEBA), with diethylenetriamine (DETA) as the curing agent.

Creating the initial MD setup to simulate DGEBA-DETA epoxy requires defining the different molecules, the force field, and the cross-linking reaction. Initially, the molecular system of interest must therefore be carefully studied, and the necessary data about the reaction precursors and other molecules must be gathered. Once the initial topology has been created, a force field must be selected to handle the atomic interactions among all of the atoms in the molecules, including those involved in the cross-linking process. This means considering the molecular structure both before and after the reaction.

**Figure 2:**
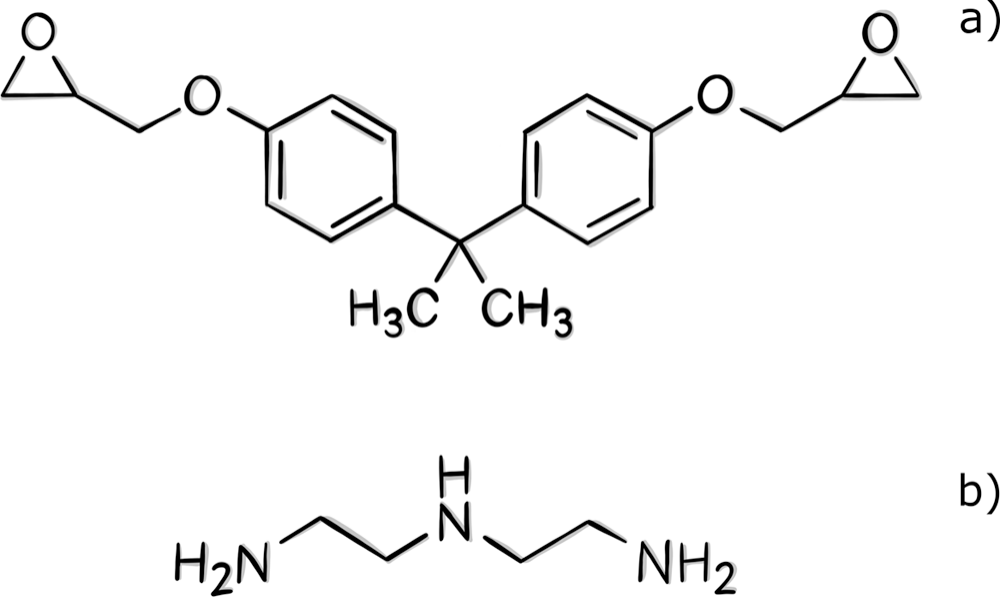
(a) Bisphenol A diglycidyl ether epoxy resin. (b) Diethylenetriamine aliphatic hardener.

Bisphenol A diglycidyl ether epoxy resin used in commercial applications (normally found as DER332 or Epon828) exhibits a weight per epoxide (WPE) in the range 172–195 g/mol/epoxy. In this study, we consider a mixture of only epoxy resin and a curing agent in a 5:2 ratio (shown in Figure 2), without the presence of other constituents. Therefore, differences in the estimated thermo-mechanical properties could be observed compared to experimental data found in the literature, as the presence of other different molecules could alter the packing process and consequently the resulting performance.

### 2.2 MD Protocol to Cure Thermosetting Resins

#### 2.2.1 Cross-Linking Protocol Overview

**Figure 3:**
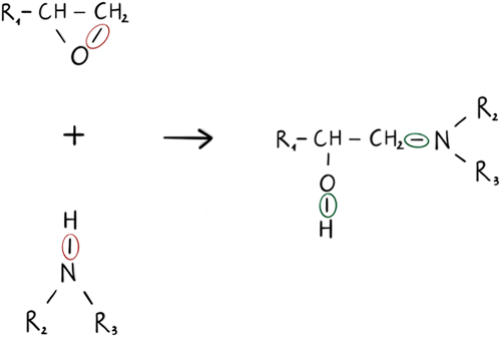
Schematic representation of the broken and created bonds in the simulation of the cross-linking process.

**Figure 4:**
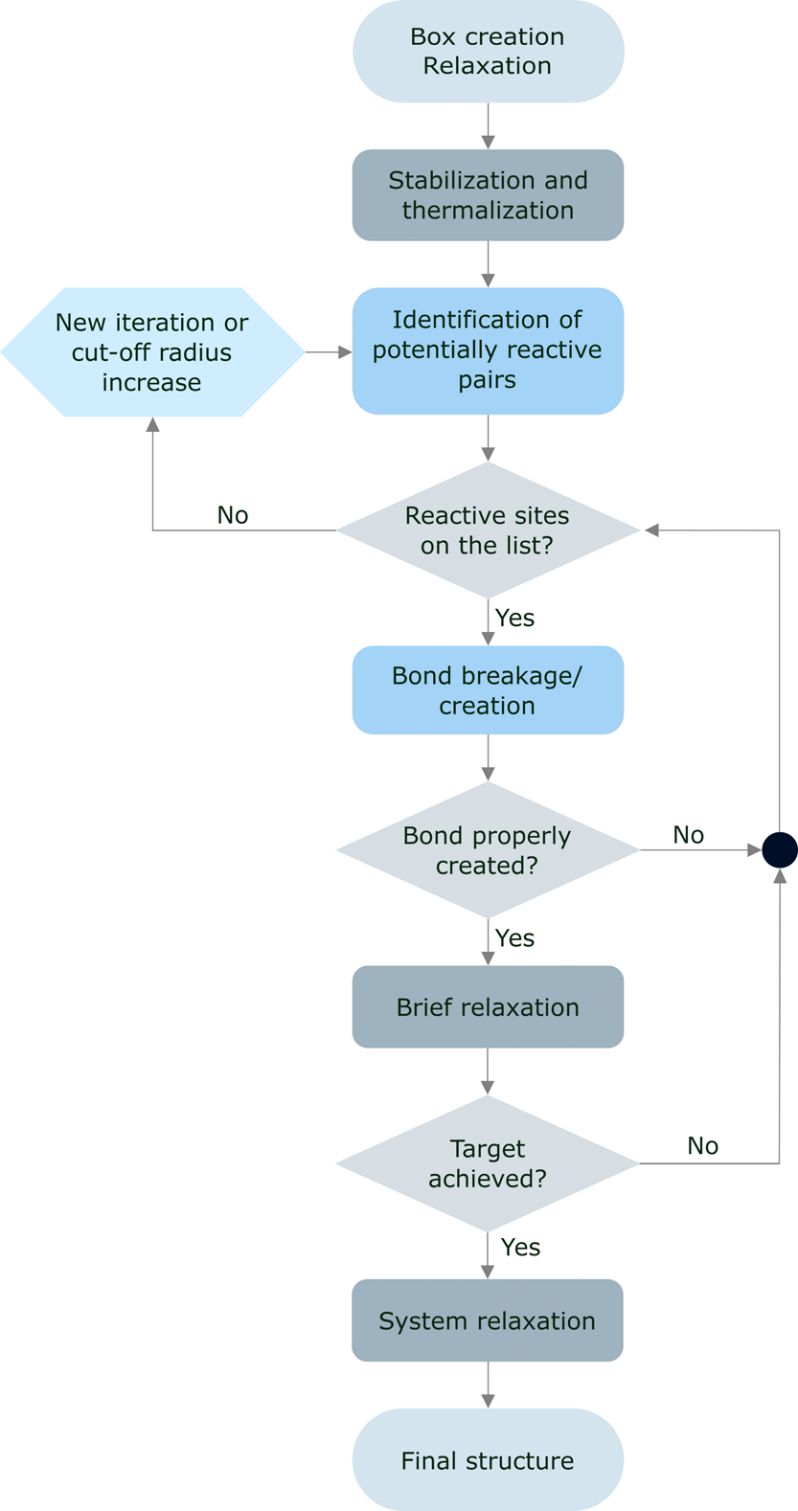
Schematic representation of the cross-linking process.

Several methods can be found in the literature to replicate the curing process of epoxy resins, as discussed in the Section 1, and the range of cross-linking degrees typically considered in MD studies for different types of resins spans from 50 to 100%. The cross-linking degrees of greatest interest from an experimental point of view generally range from 70 to 90%.^54^ In the polymerization reaction of the DGEBA-DETA epoxy, the nitrogen atoms of the amines in the DETA molecules react with the epoxy carbons in the DGEBA molecules, leading to the opening of the epoxy rings. This results in the formation of a new C–N bond between the terminal carbon of the resin and the amine nitrogen, while oxygen from the epoxy group forms a hydroxyl (−OH) group. In our approach, an initial cutoff radius *R*_cutoff_ is chosen, and each O(DGEBA)-H(DETA) pair that is at a distance less than the cutoff is iteratively made to react. The C–O and N–H bonds are broken in two successive steps. Following this, the C–N and O–H bonds are formed in two additional successive steps (Figure 3). The system is then subjected to short relaxation runs, and the cutoff radius is progressively increased. The process continues until the desired cross-linking degree, or the maximum number of iterations set, is reached. The system undergoes short intermediate relaxations to prevent the bonds created from resulting in excessive residual stresses on the system. Once the cross-linking process is finished, the resulting epoxy resin box is further relaxed before being used for other evaluations or simulations (see Figure 4).

The method implemented in this work to simulate the cross-linking process is based on approaches already present in the literature, as mentioned in the Section 1. The goal of our study is to provide a flexible, open-source solution that is easily accessible and modifiable by readers according to their specific needs. The adopted algorithm allows for the selection of different process parameters, such as the temperature at which the reaction is carried out, the equilibration times, the cutoff radius to be adopted, and so on. Multistep approaches generally alleviate network stresses, but are considered computationally expensive.^55^ The method developed here, due to the presence of numerous user-modifiable input parameters that control the breaking and formation of bonds, enables the user to select the appropriate trade-off between efficiency and accuracy to be achieved. Additionally, the code aims to help in the understanding and in-depth exploration of these processes: by tracing the cross-linking process and reconstructing it step by step within the simulation framework, it facilitates a deeper understanding of its mechanisms and implementation within a classical molecular dynamics analysis. This approach also highlights the strengths and limitations of the method, allowing users to refine and further investigate the aspects most relevant to their research.

To validate the procedure, the thermal and mechanical properties of a DGEBA-DETA cross-linked epoxy mixture generated by the method presented in this paper are studied by molecular dynamics, and the results are compared with experimental data available in the literature. In particular, thermal conductivity and elastic properties are evaluated, the former using the Müller-Plathe method^56^ and the latter by subjecting the box to slow deformation and evaluating the resulting behavior of the system.

#### 2.2.2 Curing Protocol Implementation Details

In this section, an overview of the implemented process is provided, while quantitative simulation details can be found in the next section. All simulations are carried out in LAMMPS, and handled by a self-written code in Python (see the comprehensive Zenodo archive for full details^51^).

The cross-linking process starts with an initial mixture of resin and hardener molecules; in our case, the initial computational domain consists of a mixture of DGEBA resin and DETA hardener in a 5:2 ratio (see Figure 2). In addition to the data file related to the starting mixture for cross-linking, other simulation parameters must also be provided, for example: heating temperature of the mixture, number of steps to be performed during the short intermediate relaxations, minimum and maximum reaction radius to be used, number of iterations per radius, cutoff radius increment, and so on. Moreover, some information about the reaction to be simulated must be supplied, such as the type of atoms involved, the bonds to be broken and created, and the related changes in the atomic partial charges. All the information on how to provide such parameters can be found in the help of the code made in Python and available in the Zenodo archive associated with this work,^51^ along with an example of the simulation parameters used. The large number of input parameters gives flexibility to the implemented code, allowing the process to be tuned appropriately as needed as well as to possibly adapt the process to other reactions of a similar nature by simply changing the inputs.

Initially, the parameters provided as input to the algorithm are checked to verify that they are compatible with the format requirements and that the initial topology is free of charge imbalances. At the beginning of the cross-linking process, increasing the temperature of the mixture may be useful for improving the kinetic energy of the molecules, thus facilitating the reaction. After stabilizing the initial mixture, potentially reactive sites must be identified; in our case, the search for reactive sites and the subsequent cross-linking procedure are based on the mutual positions of hydrogen H and oxygen O atoms, but a different choice could be adopted without losing generality. The search for potential reaction pairs is carried out with the help of LAMMPS, starting from the minimum cutoff radius provided as input. Once a list of pairs of reactive atoms (i.e., those that are at a shorter distance than the cutoff) has been generated and sorted in ascending order according to distance, one pair at a time is cross-linked. After one pair reacted, it is necessary to verify that the remaining reactive pairs continue to meet the distance requirements. Once the list is empty, the search for potentially reactive pairs is restarted until the maximum number of iterations set is reached. At this point, the cutoff radius is increased by the amount defined in the input parameters, and the process begins again. The cycle is repeated until the maximum cutoff radius, or desired degree of cross-linking, is reached (see Figure 4).

Short intermediate relaxations of the system are performed during the cross-linking process to avoid excessive stresses resulting from bond breakage/creation. The implemented method periodically updates the topology of the system to account for the newly formed bonds (angles, dihedrals, molecule membership, etc.), and it makes some adjustments to the box dimensions to avoid excessive anisotropic geometries. Finally, the code also manages the creation of bonds through the boundaries.

At the end of the cross-linking process, the cured system undergoes additional relaxation cycles before being used for further analyses. In our case, the computational domains are subjected to heating and cooling ramps, followed by a final relaxation.

#### 2.2.3 Molecular Dynamics Details

The MD simulations carried out with LAMMPS^47−50^ considered real units, periodic boundary conditions in all directions, and full atom style. The structure of the DGEBA and DETA molecules was built from the data provided by the PubChem database,^57^ which can be used to create the PDB files of the two precursor molecules. An initial guess composed of 800 DGEBA and 320 DETA in a cubic box with side lengths of 80 Å was created, starting from a randomly sparse configuration of molecules, with periodic boundary conditions in the three orthogonal directions. The creation of the mixture and the assignment of atom types and charges, as well as the definition of bonds, angles, and dihedrals, were handled through Packmol and VMD. Details of how this process was implemented in this case can be found in Notes S1 and S2. Note that the types of atoms, bonds, and dihedrals in the data file of the initial topology must take into account the future presence of cross-linked structures. An example of an initial data file is provided in the Zenodo archive.^51^ A Class II potential based on the COMPASS and PCFF force fields^28,58^ was adopted for the description of the types of atoms, charges, as well as the interactions between all of the elements forming the system. The main advantage of Class II force fields is the inclusion of cross-terms, which are important for accurate calculations of vibrational frequencies and for energy and structure variations (e.g., different bond lengths) in multiple conformations of a molecule. The additional energy terms do not refer to just one characteristic parameter (such as bonds, angles, dihedrals, and out-of-plane angles) but instead are functions that use combinations of them. Details of the force field parameters used can be found in Note S2 and Figure S1. The parameters are also provided as input data for simulations in LAMMPS in the Zenodo archive.^51^ Unless otherwise specified, a cutoff of 12 Å was used for nonbonded interactions, with cross-term Lennard-Jones parameters computed with sixth-power mixing rules. Long-range interactions were computed with the PPPM method with a 10^–4^ accuracy.

During the cross-linking process, the Nosé-Hoover thermostat and barostat were adopted (using the fix nvt/npt commands in LAMMPS). In our protocol, the cross-linking degree was defined as the ratio of hydrogens in amines that have reacted to the total number of potentially reactive sites at the beginning of the process. Bond breaking and creation were handled with the fix bond/break and fix bond/create commands of LAMMPS. Reaction cutoff radii in the range of 2–10 Å were used in our simulations. The update of topological information was handled by VMD, with the help of the Python code available in the Zenodo archive.^51^

An alternative approach could have included the use of the fix bond/react command already implemented in LAMMPS,^59,60^ which allows topology variations to be handled. In this case, we chose to implement a self-built multistep approach to prioritize the chance to control and modify the reaction development in the desired way and to create a flexible code that can be adapted to other needs in the future. This work is intended to be a first step in the development of an analysis protocol that could assist in deepening and increasing our understanding and study of these kinds of processes. The created code allows for targeted actions on the individual parameters of the reaction to be simulated (such as the type of atoms reacting and the bonds being broken), as well as the ways in which it occurs (such as temperature and cutoff radius), giving high flexibility to the implemented method (see Table S1).

The DGEBA-DETA epoxy resin boxes generated by the cross-linking protocol described in this paper were relaxed at 300 K and 1 atm until energy stabilization, which typically requires a simulation time of 10–20 ns. Unless otherwise specified, coupling constants of 100 timesteps and 1000 timesteps were used for the thermostat and barostat, respectively. A time step of 0.01 fs was used during the cross-linking process, as topology changes make the system unstable, and was then gradually increased to 1 fs in relaxations.

## 3 Results and Discussion

**Figure 5:**
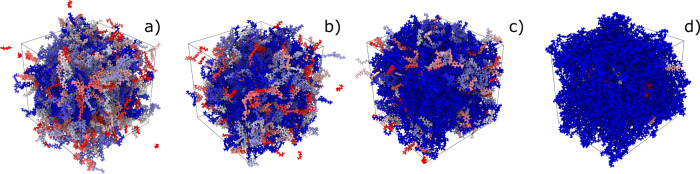
Representation of the DGEBA-DETA epoxy simulation boxes with different degrees of cross-linking: (a) 40%, (b) 50%, (c) 60%, and (d) 80%. Each molecule in the simulation box is assigned a different color.

Following the protocol described above, we produced 10 MD systems of DGEBA-DETA epoxy with different cross-linking degrees (i.e., from 0 to 90%, see Figure 5). According to the LAMMPS syntax, each molecule in the simulation box is assigned to a specific numerical molecule identifier (ID), from 1 to *n*, with *n* being the total number of molecules. In Figure 5, each molecule is represented with a different color: blue, gray, or red. During cross-linking, when DGEBA and DETA molecules are connected, the molecule ID is updated, so the total number of molecules progressively reduces. At higher degrees of cross-linking, most of the atoms belong to the same molecule; therefore, the image in Figure 5d (80% cross-linking) is almost entirely blue.

It is worth noting that the implemented cross-linking methodology allows atoms belonging to the same molecules to react; however, the code is flexible enough to possibly consider a different choice. Moreover, no distinction is made in terms of reactivity between nitrogen belonging to primary or secondary amines.^27^ Furthermore, similarly to previous works, this cross-linking algorithm does not claim to accurately represent the kinetics of the curing reaction, since the process can be accelerated or slowed down by regulating the domain temperature (and thus the thermal agitation of the atoms) and other parameters, such as the cutoff radius, thereby affecting the reaction.^27^

We tested the validity of our cross-linked numerical samples by evaluating and comparing some physical observables with experimental data in the literature such as density, thermal conductivity, and elastic modulus.

### 3.1 Density

**Figure 6:**
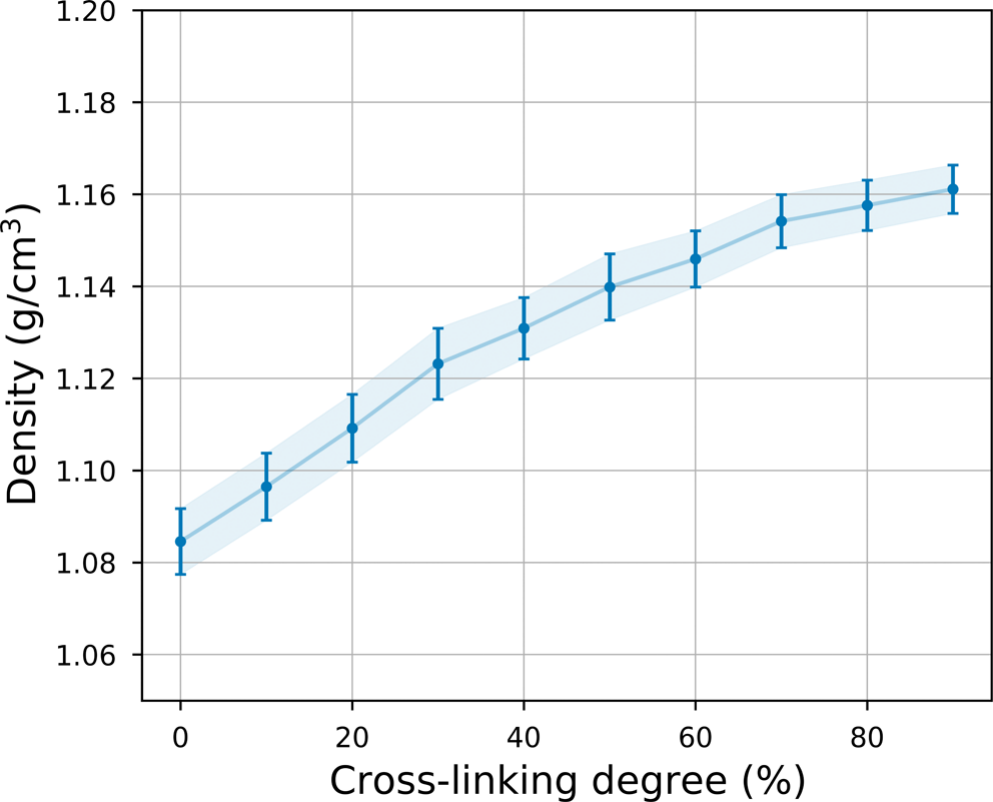
Density of DGEBA-DETA epoxy for different cross-linking degrees, in the range of 0–90%. A positive trend is observed as the cross-linking percentage increases. Each point corresponds to the average value of densities along a trajectory of 2 ns after stabilization. The error bar shows, on both sides of the average value, the difference between the maximum and minimum values of the results, divided by 2.

The density of the numerical samples was evaluated along a trajectory of 2 ns after stabilization at 300 K and 1 atm. In Figure 6 and Table S2, we summarize the results for different cross-linking degrees. The computed density ranged from 1.08 to 1.16 g/cm^3^. As expected, the higher the cross-linking degree, the denser the material. Our results agree with the experimental and numerical values observed in the literature (typically around 1.16 g/cm^3^)^20,61^ and follow the same increasing trend with increasing cross-linking degree, thus validating the structural response of our model for different cross-linking degrees.

### 3.2 Thermal Conductivity

**Figure 7:**
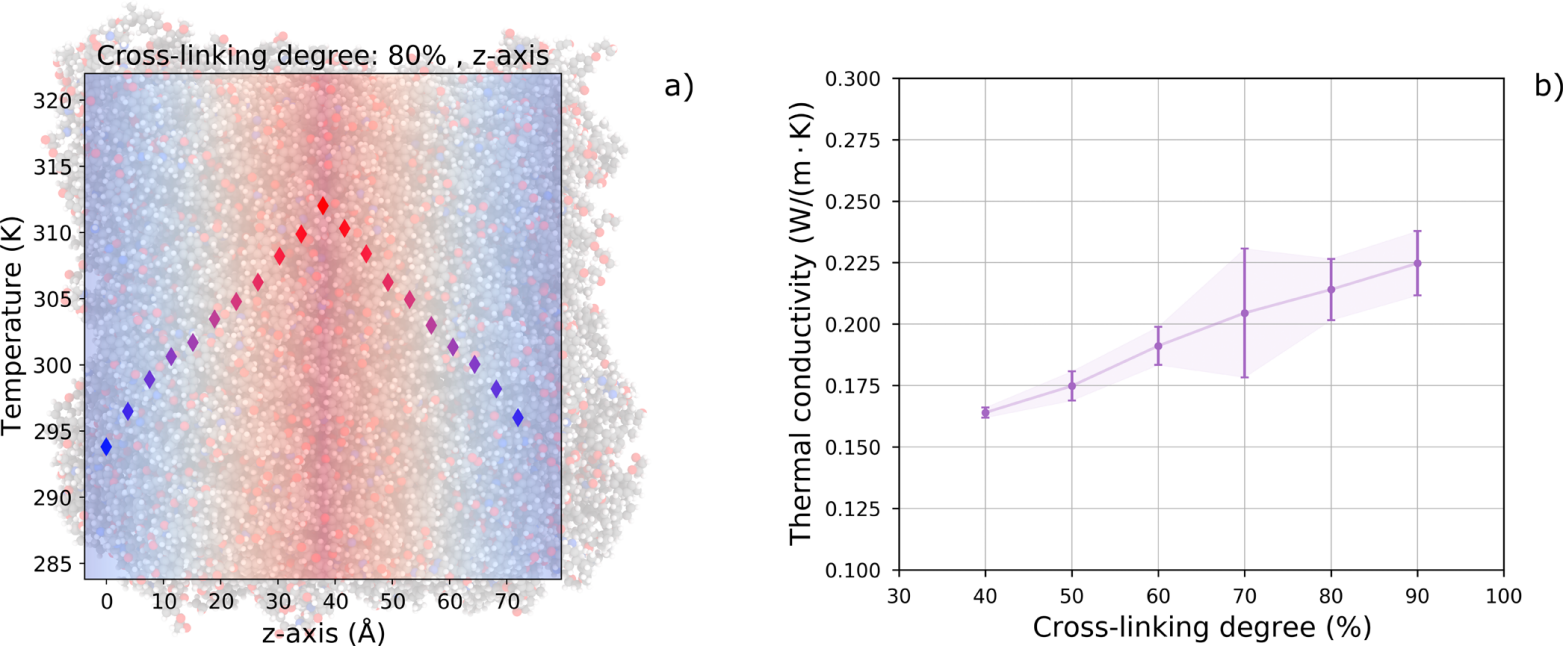
(a) Thermal gradient as created in the MD system by the Müller-Plathe algorithm. The hotter and colder regions of the simulation box are depicted in red and blue, respectively. (b) Thermal conductivity of DGEBA-DETA epoxy for different cross-linking degrees, in the range 40–90%. A positive trend is observed as the degree of curing increases. Each point corresponds to the average value of the thermal conductivities evaluated along the three orthogonal directions (i.e., *x*, *y*, and *z*). The error bar shows, on both sides of the average value, the difference between the maximum and minimum values of the results for the three directions divided by 2.

The thermal conductivity (λ) of the DGEBA-DETA epoxy domains was computed through reverse nonequilibrium molecular dynamics (rNEMD) simulations, following the Müller-Plathe^56^ algorithm as implemented in LAMMPS. This method establishes a thermal gradient in the system along one orthogonal direction (*x*, *y* or *z*) as described in Figure 7a. Full methodological details are provided in Note S3 and Figure S2, as well as in our previous work.^36^ Representative samples of the LAMMPS codes used for the thermal simulations are available in the Zenodo archive.^51^ The reported thermal conductivity values are averaged over tests performed in each orthogonal direction, while the error bar shows, on both sides of the average value, the difference between the maximum and minimum values of the results for the three directions divided by 2.

Figure 7b shows the variation of λ with the cross-linking degree. The thermal conductivity range obtained goes from 0.16 to 0.24 W/m·K. The results of thermal conductivity are summarized in Table S3. Our models perform in excellent agreement with the experimental and numerical evidence in the literature, which reports λ values around 0.2 W/m·K for similar resins; more generally, the thermal conductivity of epoxy resins ranges from 0.125 to 0.25 W/m·K, depending on the specific composition.^61−64^

### 3.3 Elastic Constants

**Figure 8:**
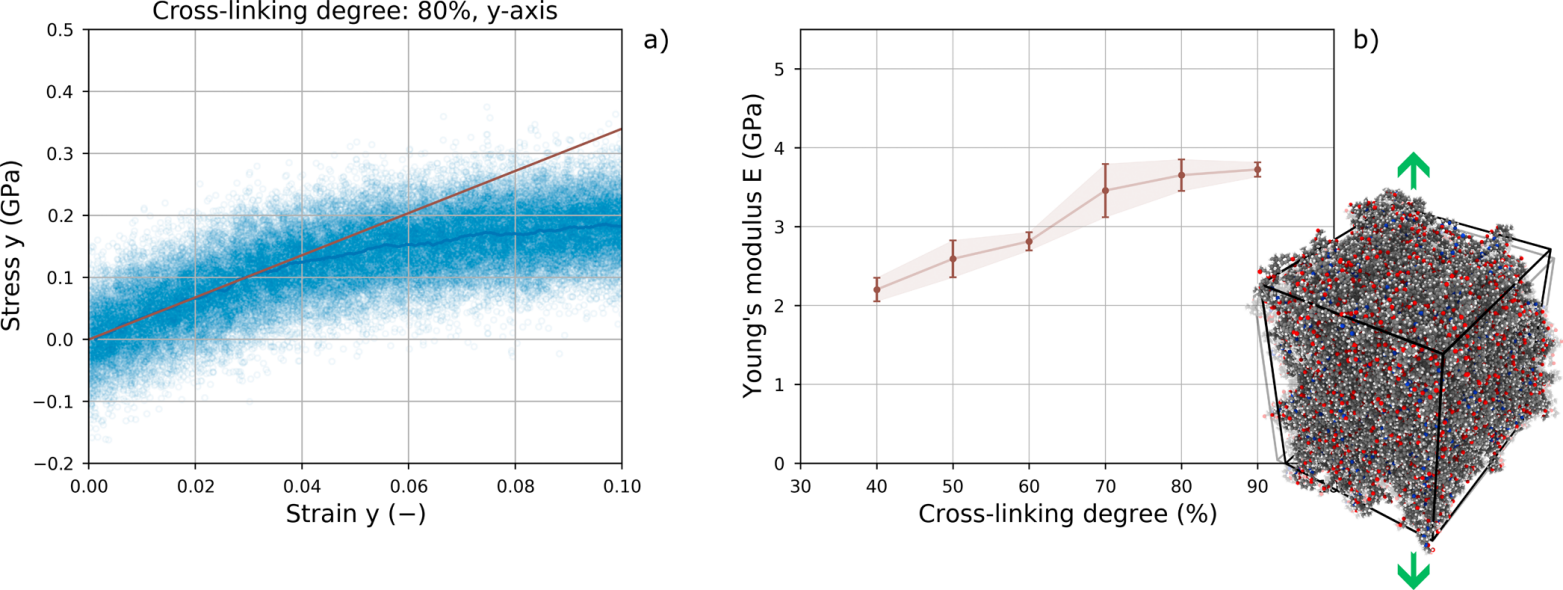
(a) Stress–strain curve obtained with nonequilibrium MD on a DGEBA-DETA epoxy domain. Dots indicate the values recorded during the deformation of the simulation box. The blue line is the moving average. The red line is the linear regression obtained between 0 and 2% deformation, assumed as the elastic region for the samples. The Young’s modulus is evaluated as the slope of the red line. (b) Young’s modulus of DGEBA-DETA epoxy for different cross-linking degrees, in the range 40–90%. A positive trend is observed as the cross-linking percentage increases. Each point corresponds to the average value of the Young’s modulus evaluated along the three orthogonal directions (i.e., *x*, *y*, and *z*). The error bars indicate the difference between the maximum and minimum values of the results for the three directions, divided by 2 and reported on both sides of the mean value.

The mechanical properties of the cross-linked polymer were assessed with nonequilibrium molecular dynamics (NEMD) simulations. The simulation box is deformed along one of the three orthogonal directions (i.e., *x*, *y*, or *z*), while the stresses acting in the same direction are recorded. Stresses and strains are then plotted as in Figure 8a, and the Young’s modulus is evaluated as the slope of the obtained curve between 0 and 2% of strain. Again, we focused our attention on six epoxy systems, with cross-linking degrees ranging from 40 to 90%. Indeed, at lower curing degrees, the observed response of the material is not fully linear, evidencing some different behavior as well.

Further details about the simulations are provided in Note S3 and Figure S3, while representative samples of the LAMMPS codes used for the simulations are available in the Zenodo archive.^51^ The values of elastic constants summarized in Figure 8 and Table S4 are the averages of the tests performed in the three orthogonal directions, while the error bars are the difference between the maximum and minimum values of the results for the three directions, divided by 2, and reported on both sides of the mean value.

Figure 8 shows the increase in the elastic modulus with the curing degree due to the more cross-linked bond network, as also reported in previous numerical and experimental results in the literature.^27^ In addition, the elastic modulus of systems with a curing degree in the range of 60–90%, which is also the most commonly adopted cross-linking degree range for several applications, is in good agreement with the experimental and numerical values reported in the literature, generally ranging from 2.4 to 5 GPa,^61,65−67^ thus further validating our proposed protocol.

The results obtained from these analyses indicate a dependence of the thermo-mechanical properties of epoxy resin on the cross-linking degree, in agreement with previous studies in the literature on the characterization of thermosetting polymers.^68^ As the degree of cross-linking increases, new bonds are created between the resin precursors and the hardener (see Figures 5 and S4), the molecular structures linked through bonded interactions grow in size, and the formation of a network leads to increased density and greater resistance to deformation (higher Young’s modulus). The thermal conductivity of epoxy resins without additional components of a different nature (e.g., carbon-based reinforcements) remains low, with an increase for high degrees of cross-linking. Previous studies have attributed the change in the thermal conductivity to the presence of a molecular network within the material, which facilitates additional pathways for heat transfer.

It is important to note that the results presented in this work have been extracted from a single epoxy sample for each degree of cross-linking, considering relatively small computational domains (on the order of 45000 atoms). To enhance the statistics, further replicas starting from different initial conditions (that can affect the reticulation of the systems and consequently the physical observables) and different combinations of input parameters should be tested. However, this evaluation goes beyond the activity of this paper, which aims to provide, describe, and test a cross-linking protocol for MD simulations based on classical force fields.

## 4 Conclusions

In this paper, we modeled the behavior of thermosetting polymers using molecular dynamics with a focus on simulating the cross-linking process of epoxy resins. Specifically, we developed a protocol that allowed us to create DGEBA-DETA epoxy resin boxes with the desired cross-linking degree and that enabled us to self-manage and flexibly handle topology changes affecting the resin during cross-linking.

Several studies in the literature have already aimed at analyzing the cross-linking process of thermosetting polymers, many of which are based on the use of commercial software or special commands developed in LAMMPS. The methodology we implemented instead focused on creating an open-source, easy-to-access code that would allow, on the one hand, the implementation of the cross-linking process and, on the other hand, a thorough understanding and direct management of the topology changes affecting the resin during the reaction. Moreover, due to the presence of numerous input parameters, it is possible to influence the evolution of the reaction in the desired way and to evaluate how temperature, number of iterations, cutoff radius, and other elements affect the process. The code was implemented in Python by exploiting open-source software and could be simply adapted to handle other molecules and similar reactions. The protocol developed as part of this work is therefore a valuable aid in the study of not only the cross-linking of epoxy resins but also other similar processes, as it allows for step-by-step tracking and direct action on variations in the topology of the simulated system. As an industrially relevant case study, 10 MD domains of DGEBA-DETA epoxy resin with cross-linking degree between 0 and 90% were generated and tested, showing density and thermo-mechanical behavior in agreement with experimental ranges found in the literature. Compared to reactive potential-based cross-linking approaches, such as Reax-FF,^17^ which would require high computational time and considerable parametrization effort to simulate chemical reactions of engineering interest,^69^ the proposed curing protocol considers the need to find an appropriate compromise between accuracy and efficiency.

The increasing focus on climate, economic, and social sustainability demands a shift toward new recycling approaches, as evidenced by several initiatives for the circularity of materials.^70^ In the future, this study could serve as a first step in developing a multiscale numerical model for the investigation and simulation of advanced recycling processes of polymeric materials.

## Data Availability

The data and scripts associated with this work are available in the Zenodo repository at the following link: https://doi.org/10.5281/zenodo.11402476.

## Supporting Information Available

Additional details on the molecular structures used, force field parameters, and cross-linking process settings, as well as the protocols for evaluating thermal conductivity and elastic properties; additional simulation results related to the assessment of thermo-mechanical properties (PDF)
