# Supplementary material for: Atomistic Modeling of Cross-Linking in Epoxy-Amine Resins: An Open-Source Protocol
Source: ACS Appl Polym Mater. 2025 Apr 3;7(8):4876–84. doi: 10.1021/acsapm.4c04208 (PMC12039963; doi:10.1021/acsapm.4c04208)
Supplement: Supplementary file 1 — ap4c04208_si_001.pdf [file ap4c04208_si_001.pdf]

# – Supporting Information –

Atomistic Modeling of Cross-Linking in Epoxy-Amine Resins: An Open-Source Protocol

Marina Provenzano,[†] Francesco Maria Bellussi,[†] Matteo Fasano,[*,†] and Hernán Chávez Thielemann[†,‡]

†*Department of Energy, Politecnico di Torino, Corso Duca degli Abruzzi 24, 10129, Torino, Italy*

‡*Department of Mechanical Engineering, Eindhoven University of Technology, 5612 AZ, Eindhoven, The Netherlands*

E-mail: matteo.fasano@polito.it

In this document, we provide supporting notes, figures and tables with additional details on model development and obtained results.

Supporting Note 1: Initial setup

To easily produce an input file for LAMMPS containing a mixture of resin and hardener, a Python script was created. This script performs all the basic calculations, then automatically runs the PACKMOL[1] software to pack all the molecules, and finally assigns all the necessary force field parameters, generating the LAMMPS file needed to run molecular dynamics simulations. The assignment and management of parameters are carried out thanks also to the support of VMD.[2] To create a solid base, the molecular structures of resin and hardener were downloaded from Pubchem,[3] in SDF or XLM format. When generating the initial mixture, attention should be paid to the correct assignment of force field parameters and consistency in defining the topology (see Supporting Figure S1).

At the end of the cross-linking process (see Supporting Table S1 for some key process parameters), the computational domain must be properly relaxed to measure properties that are stable over time during the simulation, such as density (see Supporting Table S2).

Supporting Note 2: Force Field Parameters

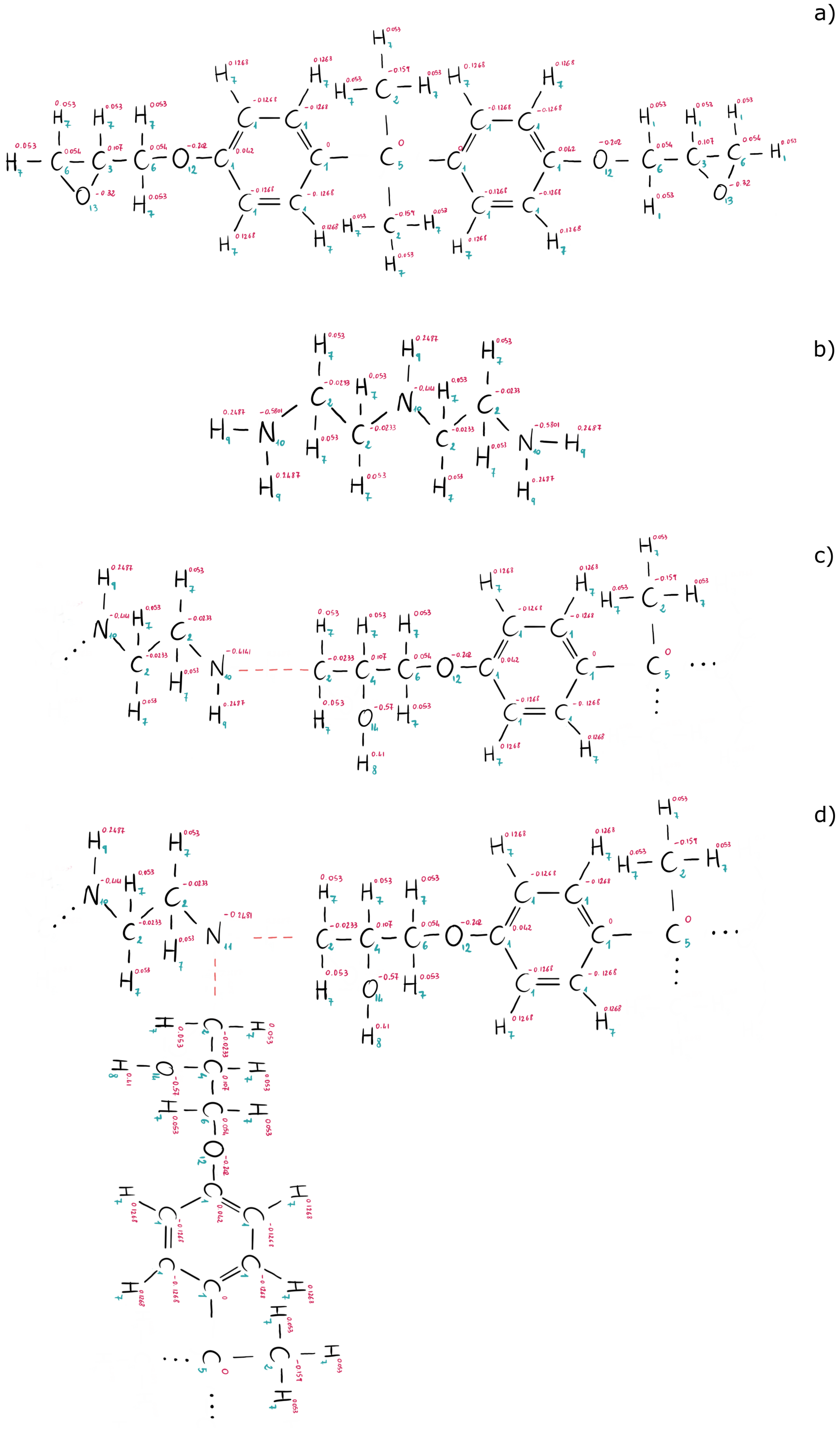

Figure S1: Schematic representation of the DGEBA and DETA molecules used for the simulations performed in this work, showing the atom IDs and the assigned charges

Masses

1 12.01115

2 12.01115

3 12.01115

4 12.01115

5 12.01115

6 12.01115

7 1.00797

8 1.00797

9 1.00797

10 14.0067

11 14.0067

12 15.9994

13 15.9994

14 15.9994

PairIJ Coeffs

1 1 0.068 3.915 12

1 2 0.0649 3.885 12

1 3 0.0521 3.885 12

1 4 0.0571 3.802 12

1 5 0.0368 3.885 12

1 6 0.0713 3.893 12

1 7 0.0271 3.574 12

1 8 0.001 3.488 12

1 9 0.0013 3.488 12

1 10 0.066 3.996 12

1 11 0.066 3.996 12

1 12 0.0796 3.671 12

1 13 0.0796 3.671 12

1 14 0.078 3.766 12

2 2 0.062 3.854 12

2 3 0.0498 3.854 12

2 4 0.055 3.768 12

2 5 0.0352 3.854 12

2 6 0.0681 3.862 12

2 7 0.0268 3.526 12

2 8 0.001 3.434 12

2 9 0.0013 3.434 12

2 10 0.0626 3.969 12

2 11 0.0626 3.969 12

2 12 0.0777 3.629 12

2 13 0.0777 3.629 12

2 14 0.0753 3.73 12

3 3 0.04 3.854 12

3 4 0.0442 3.768 12

3 5 0.0283 3.854 12

3 6 0.0547 3.862 12

3 7 0.0215 3.526 12

3 8 0.0008 3.434 12

3 9 0.0011 3.434 12

3 10 0.0503 3.969 12

3 11 0.0503 3.969 12

3 12 0.0624 3.629 12

3 13 0.0624 3.629 12

3 14 0.0605 3.73 12

4 4 0.0498 3.67 12

4 5 0.0312 3.768 12

4 6 0.0603 3.777 12

4 7 0.0265 3.386 12

4 8 0.001 3.27 12

4 9 0.0014 3.27 12

4 10 0.0543 3.895 12

4 11 0.0543 3.895 12

4 12 0.0735 3.509 12

4 13 0.0735 3.509 12

4 14 0.069 3.626 12

5 5 0.02 3.854 12

5 6 0.0387 3.862 12

5 7 0.0152 3.526 12

5 8 0.0006 3.434 12

5 9 0.0007 3.434 12

5 10 0.0356 3.969 12

5 11 0.0356 3.969 12

5 12 0.0441 3.629 12

5 13 0.0441 3.629 12

5 14 0.0428 3.73 12

6 6 0.0748 3.87 12

6 7 0.0292 3.539 12

6 8 0.0011 3.448 12

6 9 0.0014 3.448 12

6 10 0.0689 3.976 12

6 11 0.0689 3.976 12

6 12 0.0849 3.64 12

6 13 0.0849 3.64 12

6 14 0.0825 3.739 12

7 7 0.023 2.878 12

7 8 0.0015 2.565 12

7 9 0.0019 2.565 12

7 10 0.0243 3.698 12

7 11 0.0243 3.698 12

7 12 0.0484 3.124 12

7 13 0.0484 3.124 12

7 14 0.0384 3.319 12

8 8 0.008 1.087 12

8 9 0.0102 1.093 12

8 10 0.0009 3.626 12

8 11 0.0009 3.626 12

8 12 0.0022 2.941 12

8 13 0.0022 2.941 12

8 14 0.0016 3.19 12

9 9 0.013 1.098 12

9 10 0.0011 3.626 12

9 11 0.0011 3.626 12

9 12 0.0029 2.941 12

9 13 0.0029 2.941 12

9 14 0.002 3.19 12

10 10 0.065 4.07 12

10 11 0.065 4.07 12

10 12 0.0733 3.78 12

10 13 0.0733 3.78 12

10 14 0.0735 3.863 12

11 11 0.065 4.07 12

11 12 0.0733 3.78 12

11 13 0.0733 3.78 12

11 14 0.0735 3.863 12

12 12 0.12 3.3 12

12 13 0.12 3.3 12

12 14 0.1042 3.454 12

13 13 0.12 3.3 12

13 14 0.1042 3.454 12

14 14 0.096 3.58 12

Bond Coeffs

1 1.417 470.836 -627.618 1327.63

2 1.501 321.902 -521.821 572.163

3 1.0982 372.825 -803.453 894.317

4 1.3768 428.88 -738.235 1114.97

5 1.53 299.67 -501.77 679.81

6 1.53 299.67 -501.77 679.81

7 1.53 299.67 -501.77 679.81

8 1.101 345 -691.89 844.6

9 1.457 365.805 -699.637 998.484

10 1.457 365.805 -699.637 998.484

11 1.53 299.67 -501.77 679.81

12 1.101 345 -691.89 844.6

13 1.42 400.395 -835.195 1313.01

14 1.53 299.67 -501.77 679.81

15 1.101 345 -691.89 844.6

16 1.42 400.395 -835.195 1313.01

17 1.101 345 -691.89 844.6

18 1.42 400.395 -835.195 1313.01

19 1.42 400.395 -835.195 1313.01

20 0.9494 540.363 -1311.87 2132.44

21 1.006 466.74 -1073.6 1251.11

Angle Coeffs

1 118.9 61.0226 -34.9931 0

2 120.05 44.7148 -22.7352 0

3 117.94 35.1558 -12.4682 0

4 123.42 73.6781 -21.6787 0

5 111 44.3234 -9.4454 0

6 108.4 43.9594 -8.3924 -9.3379

7 102.969 38.9739 -6.2595 -8.171

8 110.77 41.453 -10.604 5.129

9 111.91 60.7147 -13.3366 -13.0785

10 111.91 60.7147 -13.3366 -13.0785

11 112.67 39.516 -7.443 -9.5583

12 110.77 41.453 -10.604 5.129

13 111.27 54.5381 -8.3642 -13.0838

14 112.67 39.516 -7.443 -9.5583

15 112.444 47.2337 -10.6612 -10.2062

16 110.954 50.8652 -4.4522 -10.0298

17 112.444 47.2337 -10.6612 -10.2062

18 110.77 41.453 -10.604 5.129

19 111.27 54.5381 -8.3642 -13.0838

20 111.27 54.5381 -8.3642 -13.0838

21 104.5 35.7454 -10.0067 -6.2729

22 110.77 41.453 -10.604 5.129

23 111.91 60.7147 -13.3366 -13.0785

24 111.91 60.7147 -13.3366 -13.0785

25 110.77 41.453 -10.604 5.129

26 111.27 54.5381 -8.3642 -13.0838

27 105.8 52.7061 -12.109 -9.8681

28 110.77 41.453 -10.604 5.129

29 112.67 39.516 -7.443 -9.5583

30 110.77 41.453 -10.604 5.129

31 111.27 54.5381 -8.3642 -13.0838

32 110.77 41.453 -10.604 5.129

33 111.27 54.5381 -8.3642 -13.0838

34 107.66 39.641 -12.921 -2.4318

35 110.62 51.3137 -6.7198 -2.6003

36 110.62 51.3137 -6.7198 -2.6003

37 108.728 58.5446 -10.8088 -12.4006

38 108.728 58.5446 -10.8088 -12.4006

39 107.66 39.641 -12.921 -2.4318

40 108.728 58.5446 -10.8088 -12.4006

41 108.728 58.5446 -10.8088 -12.4006

42 107.067 45.252 -7.5558 -9.512

BondBond Coeffs

1 68.2856 1.417 1.417

2 12.0676 1.417 1.501

3 1.0795 1.417 1.0982

4 48.4754 1.417 1.3768

5 0 0 0

6 0 0 0

7 0 0 0

8 3.3872 1.53 1.101

9 4.6217 1.53 1.457

10 4.6217 1.53 1.457

11 0 0 0

12 3.3872 1.53 1.101

13 11.4318 1.53 1.42

14 0 0 0

15 -2.1113 1.457 1.457

16 -6.4168 1.457 1.006

17 -2.1113 1.457 1.457

18 3.3872 1.53 1.101

19 11.4318 1.53 1.42

20 11.4318 1.53 1.42

21 -7.1131 1.42 1.42

22 3.3872 1.53 1.101

23 4.6217 1.53 1.457

24 4.6217 1.53 1.457

25 3.3872 1.53 1.101

26 11.4318 1.53 1.42

27 -9.6879 1.42 0.9494

28 3.3872 1.53 1.101

29 0 0 0

30 3.3872 1.53 1.101

31 11.4318 1.53 1.42

32 3.3872 1.53 1.101

33 11.4318 1.53 1.42

34 5.3316 1.101 1.101

35 12.426 1.101 1.457

36 12.426 1.101 1.457

37 23.1979 1.101 1.42

38 23.1979 1.101 1.42

39 5.3316 1.101 1.101

40 23.1979 1.101 1.42

41 23.1979 1.101 1.42

42 -1.8749 1.006 1.006

BondAngle Coeffs

1 28.8708 28.8708 1.417 1.417

2 31.0771 47.0579 1.417 1.501

3 20.0033 24.2183 1.417 1.0982

4 58.479 107.681 1.417 1.3768

5 0 0 0 0

6 0 0 0 0

7 0 0 0 0

8 20.754 11.421 1.53 1.101

9 6.0876 16.5702 1.53 1.457

10 6.0876 16.5702 1.53 1.457

11 8.016 8.016 1.53 1.53

12 20.754 11.421 1.53 1.101

13 2.6868 20.4033 1.53 1.42

14 8.016 8.016 1.53 1.53

15 -7.2229 -7.2229 1.457 1.457

16 31.8096 20.5799 1.457 1.006

17 -7.2229 -7.2229 1.457 1.457

18 20.754 11.421 1.53 1.101

19 2.6868 20.4033 1.53 1.42

20 2.6868 20.4033 1.53 1.42

21 -2.8112 -2.8112 1.42 1.42

22 20.754 11.421 1.53 1.101

23 6.0876 16.5702 1.53 1.457

24 6.0876 16.5702 1.53 1.457

25 20.754 11.421 1.53 1.101

26 2.6868 20.4033 1.53 1.42

27 28.58 18.9277 1.42 0.9494

28 20.754 11.421 1.53 1.101

29 8.016 8.016 1.53 1.53

30 20.754 11.421 1.53 1.101

31 2.6868 20.4033 1.53 1.42

32 20.754 11.421 1.53 1.101

33 2.6868 20.4033 1.53 1.42

34 18.103 18.103 1.101 1.101

35 13.4582 42.4332 1.101 1.457

36 13.4582 42.4332 1.101 1.457

37 4.6189 55.327 1.101 1.42

38 4.6189 55.327 1.101 1.42

39 18.103 18.103 1.101 1.101

40 4.6189 55.327 1.101 1.42

41 4.6189 55.327 1.101 1.42

42 28.0322 28.0322 1.006 1.006

Dihedral Coeffs

1 8.3667 0 1.2 0 0 0

2 0 0 4.4072 0 0 0

3 0 0 3.9661 0 0 0

4 0 0 4.8498 0 0 0

5 -0.2802 0 -0.0678 0 -0.0122 0

6 -0.2802 0 -0.0678 0 -0.0122 0

7 0 0 1.5 0 0 0

8 -0.1406 0 0.4168 0 0.015 0

9 -1.1506 0 -0.6344 0 -0.1845 0

10 -0.1406 0 0.4168 0 0.015 0

11 0 0 0.0316 0 -0.1681 0

12 0.7137 0 0.266 0 -0.2545 0

13 -0.6732 0 -0.4778 0 -0.167 0

14 -0.4 0 -0.4028 0 -0.245 0

15 -0.4 0 -0.4028 0 -0.245 0

16 -0.1406 0 0.4168 0 0.015 0

17 -1.1506 0 -0.6344 0 -0.1845 0

18 -0.1406 0 0.4168 0 0.015 0

19 -0.4 0 -0.4028 0 -0.245 0

20 0 0 1.559 0 0 0

21 0 0 0.0316 0 -0.1681 0

22 0.7137 0 0.266 0 -0.2545 0

23 0.7137 0 0.266 0 -0.2545 0

24 -0.4 0 -0.4028 0 -0.245 0

25 -0.6732 0 -0.4778 0 -0.167 0

26 0 0 2.35 0 0 0

27 0 0 1.7234 0 0 0

28 -0.1432 0 0.0617 0 -0.153 0

29 -0.2428 0 0.4065 0 -0.3079 0

30 -0.2428 0 0.4065 0 -0.3079 0

31 0 0 0.0316 0 -0.1681 0

32 -0.1432 0 0.0617 0 -0.153 0

33 -0.1435 0 0.253 0 -0.0905 0

34 -0.0228 0 0.028 0 -0.1863 0

35 0 0 0.0316 0 -0.1681 0

36 0.1904 0 -0.1342 0 -0.246 0

37 -0.5187 0 -0.4837 0 -0.1692 0

38 0.1904 0 -0.1342 0 -0.246 0

39 -0.1432 0 0.0617 0 -0.153 0

40 -0.1435 0 0.253 0 -0.0905 0

41 -0.1435 0 0.253 0 -0.0905 0

42 0.5302 0 0 0 -0.3966 0

43 -0.1432 0 0.0617 0 -0.153 0

44 -0.1435 0 0.253 0 -0.0905 0

45 0.1863 0 -0.4338 0 -0.2121 0

46 0.9513 0 0.1155 0 0.072 0

47 0.5302 0 0 0 -0.3966 0

48 0.3805 0 0.3547 0 -0.1102 0

49 0.3805 0 0.3547 0 -0.1102 0

50 0.1764 0 0.1766 0 -0.5206 0

51 -0.2428 0 0.4065 0 -0.3079 0

52 0 0 0 0 -0.153 0

53 0.3805 0 0.3547 0 -0.1102 0

54 0.1764 0 0.1766 0 -0.5206 0

55 -0.2428 0 0.4065 0 -0.3079 0

56 0 0 0 0 -0.153 0

57 -0.1435 0 0.253 0 -0.0905 0

58 1.1 0 -0.05 0 -0.1441 0

59 1.1 0 -0.05 0 -0.1441 0

60 -0.1435 0 0.253 0 -0.0905 0

61 1.1 0 -0.05 0 -0.1441 0

MiddleBondTorsion Coeffs

1 27.5989 -2.312 0 1.417

2 0 9.1792 0 1.417

3 0 -1.1521 0 1.417

4 0 4.8255 0 1.417

5 0 0 0 0

6 0 0 0 0

7 0 0 0 0

8 -8.0036 -7.7321 -3.064 1.457

9 -2.2208 0.5479 -0.3527 1.457

10 -8.0036 -7.7321 -3.064 1.457

11 -14.879 -3.6581 -0.3138 1.53

12 -21.8842 -7.6764 -0.6868 1.53

13 1.2472 0 0.7485 1.42

14 -5.9288 -2.7007 -0.3175 1.42

15 -5.9288 -2.7007 -0.3175 1.42

16 -8.0036 -7.7321 -3.064 1.457

17 -2.2208 0.5479 -0.3527 1.457

18 -8.0036 -7.7321 -3.064 1.457

19 -5.9288 -2.7007 -0.3175 1.42

20 0 3.9421 0 1.417

21 -14.879 -3.6581 -0.3138 1.53

22 -21.8842 -7.6764 -0.6868 1.53

23 -21.8842 -7.6764 -0.6868 1.53

24 -5.9288 -2.7007 -0.3175 1.42

25 1.2472 0 0.7485 1.42

26 0 4.8228 0 1.417

27 0 5.5432 0 1.417

28 -14.261 -0.5322 -0.4864 1.53

29 -10.4959 -0.7647 -0.0545 1.53

30 -10.4959 -0.7647 -0.0545 1.53

31 -14.879 -3.6581 -0.3138 1.53

32 -14.261 -0.5322 -0.4864 1.53

33 -16.7975 -1.2296 -0.275 1.53

34 0 0 0 0

35 -14.879 -3.6581 -0.3138 1.53

36 -6.4529 -6.8122 -1.1632 1.457

37 -3.4611 1.6996 -0.6007 1.457

38 -6.4529 -6.8122 -1.1632 1.457

39 -14.261 -0.5322 -0.4864 1.53

40 -16.7975 -1.2296 -0.275 1.53

41 -16.7975 -1.2296 -0.275 1.53

42 -6.8007 -4.6546 -1.4101 1.42

43 -14.261 -0.5322 -0.4864 1.53

44 -16.7975 -1.2296 -0.275 1.53

45 0 0.9241 -0.5889 1.42

46 0 0 0 0

47 -6.8007 -4.6546 -1.4101 1.42

48 -3.3497 1.0143 -3.0062 1.53

49 -3.3497 1.0143 -3.0062 1.53

50 -15.4174 -7.3055 -1.0749 1.53

51 -10.4959 -0.7647 -0.0545 1.53

52 0 0 0 0

53 -3.3497 1.0143 -3.0062 1.53

54 -15.4174 -7.3055 -1.0749 1.53

55 -10.4959 -0.7647 -0.0545 1.53

56 0 0 0 0

57 -16.7975 -1.2296 -0.275 1.53

58 -17.2585 -3.6157 -0.8364 1.53

59 -17.2585 -3.6157 -0.8364 1.53

60 -16.7975 -1.2296 -0.275 1.53

61 -17.2585 -3.6157 -0.8364 1.53

AngleAngleTorsion Coeffs

1 0 0 0

2 -14.4097 118.9 120.05

3 -4.8141 118.9 117.94

4 -21.0247 118.9 123.42

5 0 0 0

6 0 0 0

7 0 0 0

8 -24.3818 111.91 112.444

9 -7.5499 111.91 110.954

10 -24.3818 111.91 112.444

11 -16.164 112.67 110.77

12 -29.042 112.67 111.27

13 -12.1038 111.27 105.8

14 -19.0059 111.27 104.5

15 -19.0059 111.27 104.5

16 -24.3818 111.91 112.444

17 -7.5499 111.91 110.954

18 -24.3818 111.91 112.444

19 -19.0059 111.27 104.5

20 4.4444 120.05 117.94

21 -16.164 112.67 110.77

22 -29.042 112.67 111.27

23 -29.042 112.67 111.27

24 -19.0059 111.27 104.5

25 -12.1038 111.27 105.8

26 0.3598 117.94 117.94

27 4.2296 117.94 123.42

28 -12.564 110.77 110.77

29 -15.7572 110.77 111.91

30 -15.7572 110.77 111.91

31 -16.164 110.77 112.67

32 -12.564 110.77 110.77

33 -20.2006 110.77 111.27

34 0 0 0

35 -16.164 110.77 112.67

36 -12.5567 110.62 112.444

37 -10.4258 110.62 110.954

38 -12.5567 110.62 112.444

39 -12.564 110.77 110.77

40 -20.2006 110.77 111.27

41 -20.2006 110.77 111.27

42 -16.4438 108.728 104.5

43 -12.564 110.77 110.77

44 -20.2006 110.77 111.27

45 -10.5093 108.728 105.8

46 0 0 0

47 -16.4438 108.728 104.5

48 -11.2307 111.91 111.91

49 -11.2307 111.91 111.91

50 -27.3953 111.91 112.67

51 -15.7572 111.91 110.77

52 0 0 0

53 -11.2307 111.91 111.91

54 -27.3953 111.91 112.67

55 -15.7572 111.91 110.77

56 0 0 0

57 -20.2006 111.27 110.77

58 -14.0484 111.27 111.27

59 -14.0484 111.27 111.27

60 -20.2006 111.27 110.77

61 -14.0484 111.27 111.27

BondBond13 Coeffs

1 53 1.417 1.417

2 2.5085 1.417 1.501

3 -6.2741 1.417 1.0982

4 -2.2436 1.417 1.3768

5 0 0 0

6 0 0 0

7 0 0 0

8 0 0 0

9 0 0 0

10 0 0 0

11 0 0 0

12 0 0 0

13 0 0 0

14 0 0 0

15 0 0 0

16 0 0 0

17 0 0 0

18 0 0 0

19 0 0 0

20 0.8743 1.501 1.0982

21 0 0 0

22 0 0 0

23 0 0 0

24 0 0 0

25 0 0 0

26 -1.7077 1.0982 1.0982

27 -2.0517 1.0982 1.3768

28 0 0 0

29 0 0 0

30 0 0 0

31 0 0 0

32 0 0 0

33 0 0 0

34 0 0 0

35 0 0 0

36 0 0 0

37 0 0 0

38 0 0 0

39 0 0 0

40 0 0 0

41 0 0 0

42 0 0 0

43 0 0 0

44 0 0 0

45 0 0 0

46 0 0 0

47 0 0 0

48 0 0 0

49 0 0 0

50 0 0 0

51 0 0 0

52 0 0 0

53 0 0 0

54 0 0 0

55 0 0 0

56 0 0 0

57 0 0 0

58 0 0 0

59 0 0 0

60 0 0 0

61 0 0 0

EndBondTorsion Coeffs

1 -0.1185 6.3204 0 -0.1185 6.3204 0 1.417 1.417

2 0 -0.6918 0 0 0.2421 0 1.417 1.501

3 0 -6.8958 0 0 -0.4669 0 1.417 1.0982

4 0 0.2655 0 0 4.8905 0 1.417 1.3768

5 0 0 0 0 0 0 0 0

6 0 0 0 0 0 0 0 0

7 0 0 0 0 0 0 0 0

8 0.0997 -0.0046 -0.2657 -0.0128 -0.0495 -0.1079 1.53 1.457

9 -0.9466 0.9356 -0.5542 0.057 0.0625 0.4112 1.53 1.006

10 0.0997 -0.0046 -0.2657 -0.0128 -0.0495 -0.1079 1.53 1.457

11 0.2486 0.2422 -0.0925 0.0814 0.0591 0.2219 1.53 1.101

12 -0.319 0.4411 -0.7174 1.1538 0.8409 -0.9138 1.53 1.42

13 -0.58 0.9004 0 0 0.5343 0.9025 1.53 0.9494

14 -0.2456 1.0517 -0.7795 0.4741 1.2635 0.5576 1.53 1.42

15 -0.2456 1.0517 -0.7795 0.4741 1.2635 0.5576 1.53 1.42

16 0.0997 -0.0046 -0.2657 -0.0128 -0.0495 -0.1079 1.53 1.457

17 -0.9466 0.9356 -0.5542 0.057 0.0625 0.4112 1.53 1.006

18 0.0997 -0.0046 -0.2657 -0.0128 -0.0495 -0.1079 1.53 1.457

19 -0.2456 1.0517 -0.7795 0.4741 1.2635 0.5576 1.53 1.42

20 0 -1.797 0 0 -0.4879 0 1.501 1.0982

21 0.2486 0.2422 -0.0925 0.0814 0.0591 0.2219 1.53 1.101

22 -0.319 0.4411 -0.7174 1.1538 0.8409 -0.9138 1.53 1.42

23 -0.319 0.4411 -0.7174 1.1538 0.8409 -0.9138 1.53 1.42

24 -0.2456 1.0517 -0.7795 0.4741 1.2635 0.5576 1.53 1.42

25 -0.58 0.9004 0 0 0.5343 0.9025 1.53 0.9494

26 0 -0.689 0 0 -0.689 0 1.0982 1.0982

27 0 -1.5867 0 0 4.2641 0 1.0982 1.3768

28 0.213 0.312 0.0777 0.213 0.312 0.0777 1.101 1.101

29 0.196 0.7056 0.112 0.1022 0.209 0.6433 1.101 1.457

30 0.196 0.7056 0.112 0.1022 0.209 0.6433 1.101 1.457

31 0.0814 0.0591 0.2219 0.2486 0.2422 -0.0925 1.101 1.53

32 0.213 0.312 0.0777 0.213 0.312 0.0777 1.101 1.101

33 0.9681 0.9551 0.0436 0.5903 0.6669 0.8584 1.101 1.42

34 0 0 0 0 0 0 0 0

35 0.0814 0.0591 0.2219 0.2486 0.2422 -0.0925 1.101 1.53

36 -0.5892 0.714 0.3505 0.0628 0.0873 -0.0882 1.101 1.457

37 -1.1685 0.9266 -0.0993 0.085 0.3061 0.2104 1.101 1.006

38 -0.5892 0.714 0.3505 0.0628 0.0873 -0.0882 1.101 1.457

39 0.213 0.312 0.0777 0.213 0.312 0.0777 1.101 1.101

40 0.9681 0.9551 0.0436 0.5903 0.6669 0.8584 1.101 1.42

41 0.9681 0.9551 0.0436 0.5903 0.6669 0.8584 1.101 1.42

42 -0.6054 1.3339 0.9648 -0.162 0.1564 -1.1408 1.101 1.42

43 0.213 0.312 0.0777 0.213 0.312 0.0777 1.101 1.101

44 0.9681 0.9551 0.0436 0.5903 0.6669 0.8584 1.101 1.42

45 -1.7554 1.3145 0.2263 0.2493 0.6803 0 1.101 0.9494

46 0 0 0 0 0 0 0 0

47 -0.6054 1.3339 0.9648 -0.162 0.1564 -1.1408 1.101 1.42

48 0.0286 0.0566 -0.0493 0.0286 0.0566 -0.0493 1.457 1.457

49 0.0286 0.0566 -0.0493 0.0286 0.0566 -0.0493 1.457 1.457

50 0.0579 -0.0043 -0.1906 0.1032 0.5896 -0.4836 1.457 1.53

51 0.1022 0.209 0.6433 0.196 0.7056 0.112 1.457 1.101

52 0 0 0 0 0 0 0 0

53 0.0286 0.0566 -0.0493 0.0286 0.0566 -0.0493 1.457 1.457

54 0.0579 -0.0043 -0.1906 0.1032 0.5896 -0.4836 1.457 1.53

55 0.1022 0.209 0.6433 0.196 0.7056 0.112 1.457 1.101

56 0 0 0 0 0 0 0 0

57 0.5903 0.6669 0.8584 0.9681 0.9551 0.0436 1.42 1.101

58 1.0165 0.7553 -0.4609 1.0165 0.7553 -0.4609 1.42 1.42

59 1.0165 0.7553 -0.4609 1.0165 0.7553 -0.4609 1.42 1.42

60 0.5903 0.6669 0.8584 0.9681 0.9551 0.0436 1.42 1.101

61 1.0165 0.7553 -0.4609 1.0165 0.7553 -0.4609 1.42 1.42

AngleTorsion Coeffs

1 1.9767 1.0239 0 1.9767 1.0239 0 118.9 118.9

2 0 3.8987 0 0 -4.4683 0 118.9 120.05

3 0 2.5014 0 0 2.7147 0 118.9 117.94

4 0 10.0155 0 0 1.7404 0 118.9 123.42

5 0 0 0 0 0 0 0 0

6 0 0 0 0 0 0 0 0

7 0 0 0 0 0 0 0 0

8 -2.7883 1.5193 1.4796 1.2031 1.3645 -0.7071 111.91 112.444

9 -3.343 4.4558 -0.0346 0.2873 -0.8072 -0.096 111.91 110.954

10 -2.7883 1.5193 1.4796 1.2031 1.3645 -0.7071 111.91 112.444

11 -0.2454 0 -0.1136 0.3113 0.4516 -0.1988 112.67 110.77

12 0.5623 -0.3041 -0.4015 0.9672 -0.7566 -1.2331 112.67 111.27

13 -3.5903 2.5225 0.4888 0.8726 -0.3577 0.3888 111.27 105.8

14 -2.7466 1.4877 -0.8955 0.5676 0.945 0.0703 111.27 104.5

15 -2.7466 1.4877 -0.8955 0.5676 0.945 0.0703 111.27 104.5

16 -2.7883 1.5193 1.4796 1.2031 1.3645 -0.7071 111.91 112.444

17 -3.343 4.4558 -0.0346 0.2873 -0.8072 -0.096 111.91 110.954

18 -2.7883 1.5193 1.4796 1.2031 1.3645 -0.7071 111.91 112.444

19 -2.7466 1.4877 -0.8955 0.5676 0.945 0.0703 111.27 104.5

20 0 -0.1242 0 0 3.4601 0 120.05 117.94

21 -0.2454 0 -0.1136 0.3113 0.4516 -0.1988 112.67 110.77

22 0.5623 -0.3041 -0.4015 0.9672 -0.7566 -1.2331 112.67 111.27

23 0.5623 -0.3041 -0.4015 0.9672 -0.7566 -1.2331 112.67 111.27

24 -2.7466 1.4877 -0.8955 0.5676 0.945 0.0703 111.27 104.5

25 -3.5903 2.5225 0.4888 0.8726 -0.3577 0.3888 111.27 105.8

26 0 2.4501 0 0 2.4501 0 117.94 117.94

27 0 1.8729 0 0 2.5706 0 117.94 123.42

28 -0.8085 0.5569 -0.2466 -0.8085 0.5569 -0.2466 110.77 110.77

29 0.5111 1.6328 -1.0155 -1.1075 0.282 0.8318 110.77 111.91

30 0.5111 1.6328 -1.0155 -1.1075 0.282 0.8318 110.77 111.91

31 0.3113 0.4516 -0.1988 -0.2454 0 -0.1136 110.77 112.67

32 -0.8085 0.5569 -0.2466 -0.8085 0.5569 -0.2466 110.77 110.77

33 2.3668 2.492 -1.0122 -0.1892 0.4918 0.7273 110.77 111.27

34 0 0 0 0 0 0 0 0

35 0.3113 0.4516 -0.1988 -0.2454 0 -0.1136 110.77 112.67

36 -2.6321 0.9353 -0.8398 -1.3582 0.1465 -0.5729 110.62 112.444

37 -3.9582 2.0063 0.3213 -0.4294 -0.4442 -0.6141 110.62 110.954

38 -2.6321 0.9353 -0.8398 -1.3582 0.1465 -0.5729 110.62 112.444

39 -0.8085 0.5569 -0.2466 -0.8085 0.5569 -0.2466 110.77 110.77

40 2.3668 2.492 -1.0122 -0.1892 0.4918 0.7273 110.77 111.27

41 2.3668 2.492 -1.0122 -0.1892 0.4918 0.7273 110.77 111.27

42 -1.8234 1.6393 0.5144 -0.7777 0.434 -0.6653 108.728 104.5

43 -0.8085 0.5569 -0.2466 -0.8085 0.5569 -0.2466 110.77 110.77

44 2.3668 2.492 -1.0122 -0.1892 0.4918 0.7273 110.77 111.27

45 -3.406 1.6396 0.0737 0 -0.281 -0.5944 108.728 105.8

46 0 0 0 0 0 0 0 0

47 -1.8234 1.6393 0.5144 -0.7777 0.434 -0.6653 108.728 104.5

48 1.3673 0.4528 -2.77 1.3673 0.4528 -2.77 111.91 111.91

49 1.3673 0.4528 -2.77 1.3673 0.4528 -2.77 111.91 111.91

50 2.0125 0.944 -2.7612 -1.9225 -1.345 0.221 111.91 112.67

51 -1.1075 0.282 0.8318 0.5111 1.6328 -1.0155 111.91 110.77

52 0 0 0 0 0 0 0 0

53 1.3673 0.4528 -2.77 1.3673 0.4528 -2.77 111.91 111.91

54 2.0125 0.944 -2.7612 -1.9225 -1.345 0.221 111.91 112.67

55 -1.1075 0.282 0.8318 0.5111 1.6328 -1.0155 111.91 110.77

56 0 0 0 0 0 0 0 0

57 -0.1892 0.4918 0.7273 2.3668 2.492 -1.0122 111.27 110.77

58 0.5511 0.9737 -0.6673 0.5511 0.9737 -0.6673 111.27 111.27

59 0.5511 0.9737 -0.6673 0.5511 0.9737 -0.6673 111.27 111.27

60 -0.1892 0.4918 0.7273 2.3668 2.492 -1.0122 111.27 110.77

61 0.5511 0.9737 -0.6673 0.5511 0.9737 -0.6673 111.27 111.27

Table S1: Example of some key parameters that can be acted upon to change the evolution of the cross-linking reaction implemented through Python code. More information can be found in the Zenodo archive.[4]

Minimum reaction radius	2.0	Å
Maximum reaction radius	10.0	Å
Radius step growth	0.25	Å
Iterations per radius	10	-
Cross-linking degree	45	%
Oven temperature	500	K
Simulation time step	0.01	fs
NVT steps per bond	1000	-
NPT steps per bond	1000	-
Stopping tolerance	1.0e-8	-
LJ cut-off	12	Å

Supporting Note 3: Physical Observables

Thermal conductivity

The thermal conductivity of the epoxy resin systems is evaluated through reverse non-equilibrium molecular dynamics (RNEMD) following Müller-Plathe's approach.[5] In this approach, the simulation box is divided into N even bins, along one orthogonal direction (say, x). Then, a heat flux is imposed on the system. In the RNEMD method, the energy is continuously and artificially transferred - thus creating the heat flux -, from the cold region (the first bin of the simulation domain) to the hot region (corresponding to the central bin of the simulation domain), by exchanging the velocity of the hottest atom in the cold region with the velocity of the coldest atom in the hot region.[5] The imposed artificial heat flux ($\boldsymbol{J_x}$) from the cold to the hot region can be computed as:

$$\boldsymbol{J_x} = \frac{1}{2tA} \sum_{transfer} \frac{m}{2} \left(v_{hot}^2 - v_{cold}^2 \right), \tag{S1}$$

where t is the simulation time, A is the cross-sectional area perpendicular to the heat flux direction, and m is the mass of atoms, with the summation done over all the velocity exchange processes. $\boldsymbol{J_x}$ generates a temperature gradient ($\frac{\partial T}{\partial x_i}$) throughout the system, and the corresponding thermal conductivity λ can be then calculated using Fourier's law (see Figure 7 of the main manuscript, and Supporting Figure S2):

$$\lambda = \lim_{\frac{\partial T}{\partial x_i} \to 0} \lim_{t \to \infty} - \frac{\langle \boldsymbol{J_x}(t) \rangle}{\left\langle \frac{\partial T}{\partial x_i} \right\rangle}. \tag{S2}$$

The procedure is replicated along the three orthogonal directions (x, y, z). Thus, the results reported in Figure 7 of the main manuscript and summarized in Supporting Table S3 are the averaged values over the three directions. The error bar shows, on both sides of the average value, the difference between the maximum and minimum values of the results for the three directions, divided by two.

Elastic constants

The elastic properties of the epoxy resin systems are evaluated through non-equilibrium molecular dynamics (NEMD) simulations. We applied a box deformation along one orthogonal direction (say x) while leaving the other two (y, z) free to relax (see Figure 8 of the main manuscript). This process was implemented by applying a thermostat at the equilibrium temperature (300 K) and a barostat at 0 atm in the two directions y, z orthogonal to the deformation x. This allowed us to compute not only the stress-strain curve and thus Young's modulus (E), but also the transverse strain associated with the shrinkage on the two directions orthogonal to the applied deformation ($\epsilon_{y,z} - \epsilon_x$), and thus the Poisson ratio (ν). A strain rate of $5 \cdot 10^{-7}\text{fs}^{-1}$ was chosen, a value in agreement with what has been used in other studies in the literature.[6] Tests were also performed at different strain rates, and it was verified that we were in an area where the elastic modulus value at high cross-linking degrees was independent of the strain rate used. The elastic modulus is evaluated considering the slope of the linear regression of the initial portion of the stress-strain curve (between 0 and 0.02 strain, see Figure 8 of the main manuscript). Similarly, the Poisson ratio is evaluated considering the slope of the linear regression of the initial portion of the strain-strain curve (between 0 and 0.02 strain, see Supporting Figure S3). Finally, under the assumption of isotropic material, the transverse elastic modulus (G) is evaluated as:

$$G = \frac{E}{2(1+\nu)}. \tag{S3}$$

The procedure is replicated along the three orthogonal directions (x, y, z). Thus, the results reported in Figure 8b of the main manuscript and summarized in Supporting Table S4 are the averaged values over the three directions. The error bar shows, on both sides of the average value, the difference between the maximum and minimum values of the results for the three directions, divided by two.

Supporting Figures: Additional Results

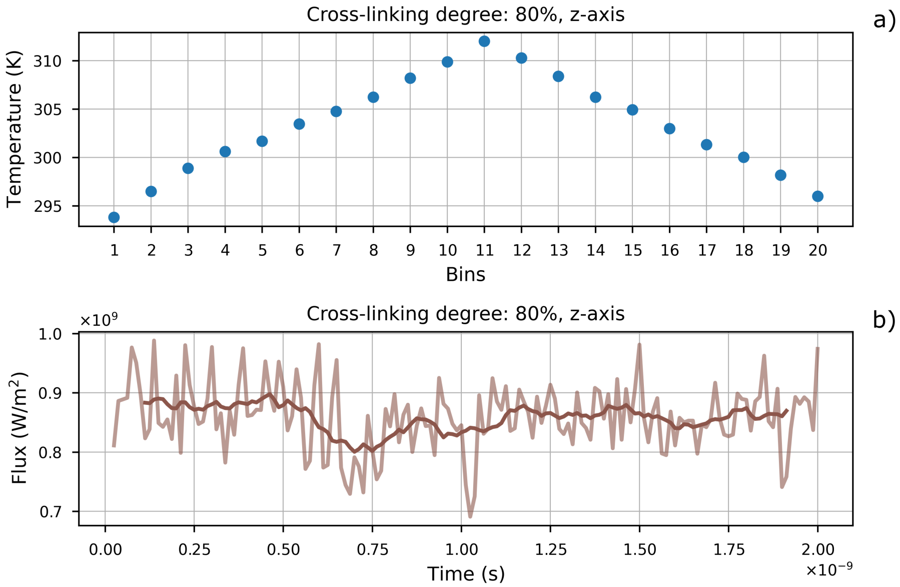

Figure S2: a) Temperature profile inside the box during the thermal conductivity evaluation process; cross-linking degree of 80%, z-direction. b) Heat flux exchanged during the simulation for thermal conductivity evaluation; cross-linking degree of 80%, z-direction.

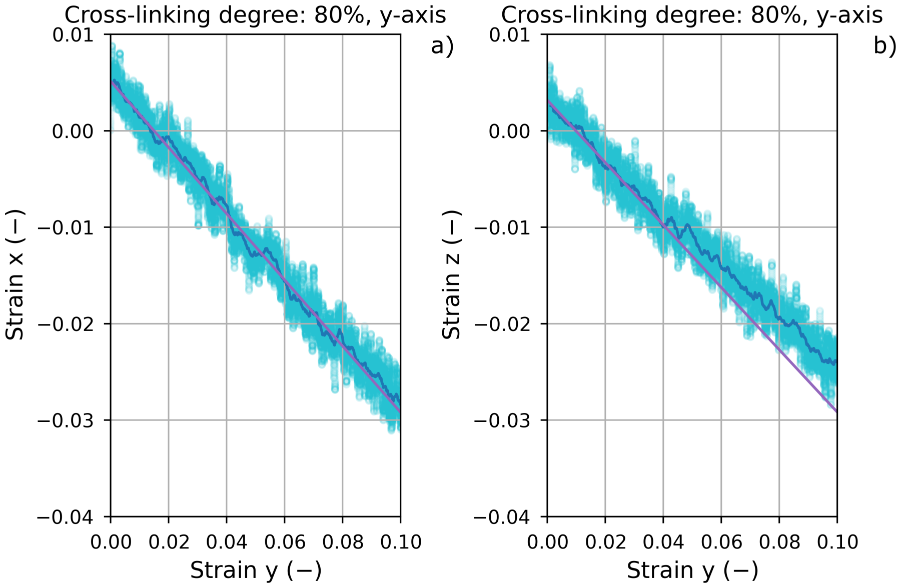

Figure S3: a) Strain y - strain x diagram and b) strain y - strain z diagram when evaluating elastic properties. Cross-linking degree of 80%, y-direction.

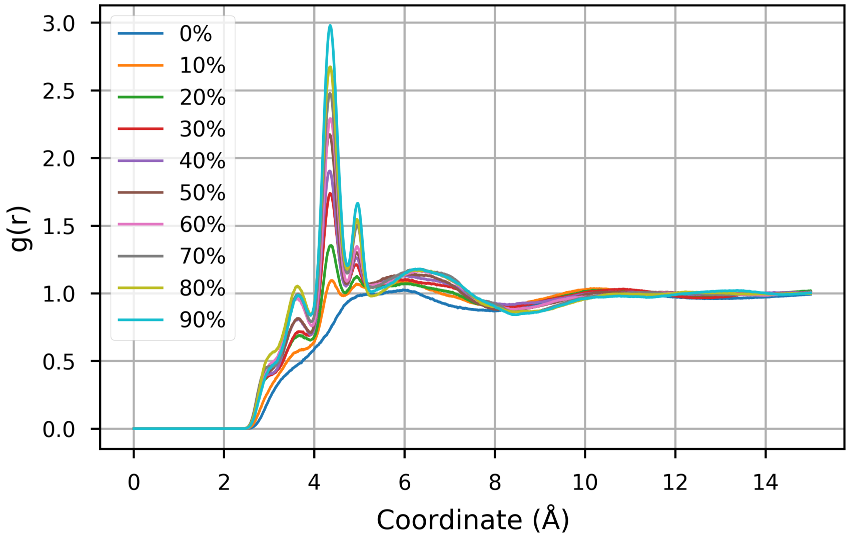

Figure S4: Radial distribution function evaluated with LAMMPS[7–10] for oxygen atoms (O) with atom type 12 and nitrogen atoms (N) with atom types 10 and 11 (see Supporting Figure S1), for ten different boxes with varying degrees of cross-linking. As the cross-linking degree increases, the number of bonds between resin precursors and hardener also increases, leading to a change in the associated radial distribution function.

Supporting Tables: Additional Results

Table S2: Density (ρ, kg/m^3) of the cross-linked epoxy resin boxes tested in this study; 300 K, 1 atm. The density of numerical samples was evaluated along the last 2 ns of trajectory. The error indicates the difference between the maximum and minimum values of the results, divided by two. The standard deviation (σ) for the data on which the mean value was calculated is also reported.

	0%	10%	20%	30%	40%	50%	60%	70%	80%	90%
ρ (kg/m^3)	1085	1096	1109	1123	1131	1140	1146	1154	1158	1161
$Err(\rho)$ (kg/m^3)	7.14	7.30	7.37	7.73	6.67	7.18	6.10	5.77	5.46	5.26
σ (kg/m^3)	2.27	2.22	2.23	2.06	1.99	1.88	1.85	1.73	1.68	1.59

Table S3: Thermal conductivity (k, W/m·K) of the cross-linked epoxy resin boxes tested in this study. Each value corresponds to the average value of the estimations made along the three orthogonal directions (*i.e.*, x, y, and z). The error indicates the difference between the maximum and minimum values of the results for the three directions, divided by two.

	40%	50%	60%	70%	80%	90%
k (W/m·K)	0.164	0.175	0.191	0.205	0.214	0.225
$Err(k)$ (W/m·K)	0.002	0.006	0.008	0.026	0.012	0.013

Table S4: Young's modulus (E, GPa), shear modulus (G, GPa) and Poisson's ratio (ν) of the cross-linked epoxy resin boxes tested in this study. Each value corresponds to the average value of the estimations made along the three orthogonal directions (*i.e.*, x, y, and z). The error indicates the difference between the maximum and minimum values of the results for the three directions, divided by two.

	40%	50%	60%	70%	80%	90%
E (GPa)	2.20	2.59	2.81	3.46	3.65	3.72
$Err(E)$ (GPa)	0.15	0.23	0.12	0.34	0.20	0.09
G (GPa)	0.84	0.99	1.06	1.30	1.37	1.40
$Err(G)$ (GPa)	0.06	0.10	0.05	0.16	0.08	0.03
ν (-)	0.32	0.30	0.32	0.33	0.33	0.33
$Err(\nu)$ (-)	0.01	0.03	0.01	0.03	0.01	0.01

References

(1) Martinez, L.; Andrade, R.; Birgin, E. G.; Martínez, J. M. PACKMOL: A package for building initial configurations for molecular dynamics simulations. *J. Comput. Chem.* **2009**, *30*, 2157–2164, DOI: 10.1002/jcc.21224.

(2) Humphrey, W.; Dalke, A.; Schulten, K. VMD – Visual Molecular Dynamics. *J. Mol. Graphics* **1996**, *14*, 33–38, DOI: 10.1016/0263-7855(96)00018-5.

(3) Kim, S.; Thiessen, P. A.; Bolton, E. E.; Chen, J.; Fu, G.; Gindulyte, A.; Han, L.; He, J.; He, S.; Shoemaker, B. A.; Wang, J.; Yu, B.; Zhang, J.; Bryant, S. H. PubChem Substance and Compound databases. *Nucleic Acids Res.* **2016**, *44*, D1202–13, DOI: 10.1093/nar/gkv951.

(4) Provenzano, M.; Bellussi, F. M.; Fasano, M.; Chávez Thielemann, H. Data for Atomistic Modeling of Cross-Linking in Epoxy-Amine Resins: An Open-Source Protocol. Zenodo. **2024**, DOI: 10.5281/zenodo.11402476.

(5) Müller-Plathe, F. A simple nonequilibrium molecular dynamics method for calculating the thermal conductivity. *J. Chem. Phys.* **1997**, *106*, 6082–6085, DOI: 10.1063/1.473271.

(6) Li, C.; Strachan, A. Molecular dynamics predictions of thermal and mechanical properties of thermoset polymer EPON862/DETDA. *Polymer* **2011**, *52*, 2920–2928, DOI: 10.1016/j.polymer.2011.04.041.

(7) Plimpton, S. Fast Parallel Algorithms for Short – Range Molecular Dynamics. *J. Comput. Phys.* **1995**, *117*, 1–19, DOI: 10.1006/jcph.1995.1039.

(8) Thompson, A. P.; Aktulga, H. M.; Berger, R.; Bolintineanu, D. S.; Brown, W. M.; Crozier, P. S.; in 't Veld, P. J.; Kohlmeyer, A.; Moore, S. G.; Nguyen, T. D.; Shan, R.; Stevens, M. J.; Tranchida, J.; Trott, C.; Plimpton, S. J. LAMMPS - a flexible simulation tool for particle-based materials modeling at the atomic, meso, and continuum scales. *Comput. Phys. Commun.* **2022**, *271*, 108171, DOI: 10.1016/j.cpc.2021.108171.

(9) Brown, W. M.; Wang, P.; Plimpton, S. J.; Tharrington, A. N. Implementing molecular dynamics on hybrid high performance computers–short range forces. *Comput. Phys. Commun.* **2011**, *182*, 898–911, DOI: 10.1016/j.cpc.2010.12.021.

(10) Brown, W. M.; Kohlmeyer, A.; Plimpton, S. J.; Tharrington, A. N. Implementing molecular dynamics on hybrid high performance computers – Particle–particle particle-mesh. *Comput. Phys. Commun.* **2012**, *183*, 449–459, DOI: 10.1016/j.cpc.2011.10.012.
